# Effects of cyclin D_1 _gene amplification and protein expression on time to recurrence in postmenopausal breast cancer patients treated with anastrozole or tamoxifen: a TransATAC study

**DOI:** 10.1186/bcr3161

**Published:** 2012-04-04

**Authors:** Katja Lundgren, Matthew Brown, Silvia Pineda, Jack Cuzick, Janine Salter, Lila Zabaglo, Anthony Howell, Mitch Dowsett, Göran Landberg

**Affiliations:** 1 Center for Molecular Pathology, Department of Laboratory Medicine, Lund University, Malmö University Hospital, SE-205 02 Malmö, Sweden; 2 Breakthrough Breast Cancer Research Unit, School of Cancer, Enabling Sciences and Technology, University of Manchester, Manchester Academic Health Science Centre Paterson Institute for Cancer Research, The Christie NHS Foundation Trust, Wilmslow Road, Manchester M20 4BX, UK; 3 Cancer Research UK Centre for Epidemiology, Mathematics and Statistics, Queen Mary University of London, Wolfson Institute of Preventive Medicine, London EC1M 6BQ, UK; 4 Breakthrough Breast Cancer Research Centre, Institute of Cancer Research, 237 Fulham Road, London SW3 6JB, UK; 5 Royal Marsden Hospital, 237 Fulham Road, London SW3 6JJ, UK; 6 Sahlgrenska Cancer Center, University of Gothenburg, 405 30 Göteborg, Sweden

## Abstract

**Introduction:**

Gene amplification of *CCND1 *is observed in a subgroup of breast cancers with poor prognosis, whereas overexpression of the protein cyclin D_1 _has been linked to both worse and better clinical outcome. *CCND1 *amplification and protein overexpression have also been associated with resistance to treatment with tamoxifen or even to a potentially detrimental effect of tamoxifen.

**Methods:**

To clarify these challenging and partly contrasting treatment predictive and prognostic links for cyclin D_1 _we analysed a large cohort of postmenopausal breast cancer patients randomised to receive either adjuvant anastrozole or tamoxifen, as part of the Arimidex, Tamoxifen, Alone or in Combination (ATAC) trial. The *CCND1 *amplification status and protein expression of cyclin D_1 _were assessed by chromogenic *in situ *hybridisation and immunohistochemistry, respectively, in 1,155 postmenopausal, oestrogen-receptor-positive breast cancer patients included in the TransATAC substudy.

**Results:**

Amplification of *CCND1 *was observed in 8.7% of the tumours and was associated with increased risk of disease recurrence (hazard ratio = 1.61; 95% confidence interval, 1.08 to 2.41) after adjustment for other clinicopathological parameters. In contrast, nuclear expression of cyclin D_1 _protein was associated with decreased recurrence rate (hazard ratio = 0.6; 95% confidence interval, 0.39 to 0.92). The intensity of nuclear or cytoplasmic expression was not of prognostic value. There was no significant interaction between cyclin D_1 _status and treatment efficacy, ruling out any major detrimental effect of tamoxifen in *CCND1*-amplified postmenopausal breast cancer.

**Conclusions:**

In summary, *CCND1 *amplification and low nuclear expression of cyclin D_1 _predicted poor clinical outcome in postmenopausal breast cancer patients treated with either anastrozole or tamoxifen.

**Trial Registration:**

Current Controlled Trials ISRCTN18233230.

## Introduction

Hormone dependence is a fundamental hallmark of the majority of breast cancers, and tumour growth can be inhibited either by deprivation of circulating oestrogens or by antagonising the effect of these hormones on their receptors [1]. The selective oestrogen receptor (ER) modulator tamoxifen has long been the most commonly used adjuvant therapy for patients with advanced hormone-sensitive breast cancer [2]. In recent years, however, aromatase inhibitors have become an alternative treatment option for postmenopausal women with breast cancer. An aromatase inhibitor acts by interfering with the enzyme that converts androgens to oestrogen, and reduces tumour and systemic oestrogen concentration [3]. The third-generation selective aromatase inhibitor anastrozole (Arimidex) reduces serum oestradiol to nanomolar concentrations [4]. The Arimidex, Tamoxifen, Alone or in Combination (ATAC) trial was designed to compare the efficacy of anastrozole alone or in combination with the established adjuvant treatment, tamoxifen for 5 years, as adjuvant treatment for postmenopausal women with operable breast cancer [5]. The study demonstrated that the efficacy of anastrozole was higher compared with tamoxifen alone, and also superior to the combination of both agents [5,6]. After a median follow-up of 10 years, 5 years after completion of treatment, the significant advantage for anastrozole over tamoxifen as initial adjuvant therapy for postmenopausal, ER-positive breast cancer patients was confirmed [7].

In breast cancer, genetic alterations such as amplifications and deletions occur within the tumour at high frequencies, and a number of these alterations are closely related to poor clinical outcome. One such region of amplification is 11q13, harbouring the cyclin D_1 _gene *CCND1 *[8-10]. Cyclin D_1 _plays a crucial role as a cell cycle regulator, promoting progression through the G_1_-S phase, following complex formation with CDK4/6 and phosphorylation of the retinoblastoma protein [11]. Various studies have described the oncogenic capacity of cyclin D_1 _*in vitro*, and overexpression *in vivo *results in tumour formation [12-14]. Overexpression of cyclin D_1 _is observed in approximately 50% of breast cancers [15,16], and cyclin D_1 _is one of the most commonly overexpressed proteins in this form of cancer. A number of studies report cyclin D_1 _overexpression to be a predictor of worse prognosis [17,18], while others have found an association with an ER-positive phenotype and a better clinical outcome [19-23]. In about 15% of all primary breast cancers, overexpression is due to amplification of the corresponding gene *CCND1 *[15,24,25], and this specific amplification has been linked to poor prognosis [23,26].

Despite the presence of ERα, approximately 50% of breast cancers develop resistance to hormonal treatment, a major clinical limitation of breast cancer therapy [27,28]. The mechanisms behind this phenomenon have been extensively studied, and imply a complex signalling network governing ER function and interaction with various co-regulators [29-32]. Cyclin D_1 _is one such co-factor, known to interact with ERα and, independently of oestrogen, activate the receptor and potentially modify oestrogen/anti-oestrogen responses [33,34]. Overexpression of cyclin D_1 _has been reported to result in a conformational change in ERα that induces receptor activation in the presence of the novel selective ER modulator arzoxifene, which in turn promotes growth of MCF-7 cells - indicating a change from antagonist to agonist [28]. This study also suggests that different mechanisms are required to confer resistance depending on the specific anti-oestrogen administered, and that changes in the conformation of ERα play a crucial role in anti-hormonal insensitivity. A similar study demonstrated that overexpression of cyclin D_1 _reversed the growth inhibitory effect of tamoxifen in two ER-positive breast cancer cell lines [35]. In line with these experimental findings we have previously observed that cyclin D_1 _overexpression was associated with tamoxifen resistance in premenopausal and postmenopausal breast cancer [21,36]. Worryingly, amplification of *CCND1 *was further linked to a potentially detrimental effect of tamoxifen in premenopausal breast cancer patients, when compared with randomised control patients not receiving any adjuvant therapy [36].

The aim of our study was to characterise the association between *CCND1 *amplification and cyclin D_1 _protein expression and breast cancer recurrence in a large randomised cohort of postmenopausal patients with ER-positive breast cancer treated with endocrine therapy. In addition, we aimed to assess whether there was a significant difference in response to anastrozole versus tamoxifen according to cyclin D_1 _gene and protein status, and thereby to address any potentially unfavourable effects of tamoxifen in subgroups of breast cancer defined by *CCND1 *amplification.

## Materials and methods

### Patients

The ATAC trial originally evaluated the efficacy and safety of 5 years of anastrozole, tamoxifen, or the combination of both treatments in postmenopausal patients presenting with localised breast cancer [7]. For the TransATAC protocol, formalin-fixed, paraffin-embedded blocks of the primary tumour were collected from as many hormone-receptor-positive patients as possible, from the monotherapy trial arms [37]. The endpoint for the analyses was any breast cancer recurrence and the median follow-up time was 10 years. The original study was performed according to the Declaration of Helsinki after approval by an institutional review board and ethics committee, and informed consent was obtained from all patients enrolled in the study.

### Immunohistochemistry

Nine tissue microarrays (TMAs) were used, each originally including from 165 to 200 tumour tissue samples from the patients included in the TransATAC study. This set of TMAs had one tissue core for each patient: a sample set was analysed for cyclin D_1 _protein expression and a set was analysed for *CCND1 *copy number. For detailed description of the TMA assembly we refer to our previously published study [37]. The TMA slides were deparaffinised, rehydrated and microwave-treated in target retrieval solution pH 9.9 (Dako, Glostrup, Denmark), and were processed in an automated immunostainer (Techmate 500; Dako, Copenhagen, Denmark) using the Envision software (Dako, Glostrup, Denmark). The antibody employed was a mouse monoclonal antibody reactive against human cyclin D_1 _(1:100, clone DSC-6; Dako, Glostrup, Denmark).

Staining of cyclin D_1 _was assessed as cytoplasmic staining intensity (0 to 2) as well as nuclear staining intensity (0 to 3) and fraction-positive nuclei (0, < 1%, 1 to 9%, 10 to 32%, 33 to 67% and > 67%) according to the Allred Score [38]. Evaluation was performed by two independent observers (one a pathologist), with the pathologist's score superseding the other observer's at consolidation. Conflicting observations were low (< 5%) for all evaluations made. All immunohistochemical evaluations were performed without knowledge of tumour characteristics. In cases of no evaluation, the tumour cores were either nonrepresentative (that is, no invasive tumour cells) or were missing. This study was carried out and is reported according to REMARK guidelines [39].

### Chromogenic *in situ *hybridisation

Chromogenic *in situ *hybridisation (CISH) was performed according to the Zymed SPoT-Light Cyclin D_1 _Probe protocol suited for CISH [40] using the SPoT-Light Cyclin D_1 _Amplification Probe (Zymed Laboratories, Invitrogen Immunodetection, San Francisco, CA, USA). Pretreatment procedures included heating and enzyme digestion to optimise the CISH performance. Nonamplified cases were classified as 0, cases with up to 8 copies classified as 1 and > 8 copies classified as 2. In statistical analyses, classifications 1 and 2 were both included in the subgroup defined as amplified.

### Statistical analyses

The primary endpoint for the analyses was time to recurrence (TTR), also known as the recurrence-free interval. TTR was defined as the time from randomisation to first locoregional recurrence, distant recurrence or contralateral disease. Statistical analyses were performed according to a prespecified statistical analysis plan approved by the ATAC Steering Committee. Cox proportional hazards regression models were fitted to TTR, and hazard ratios (HRs) and associated 95% confidence intervals (CIs) were estimated. The statistical tests employed for correlations between cyclin D_1 _variables and clinicopathological parameters were the Armitage's trend test, the Wilcoxon test, the Goodman's test and the Cuzick test [41]. Multiple hypothesis testing was not corrected for, thus marginal *P *values should be interpreted with caution. The contribution of cyclin D_1 _protein expression was analysed by the change in likelihood ratio chi-squared test (one degree of freedom) univariately and multivariately, in addition to a model with tumour size, nodal status, grade (central) and Ki67 expression, for all patients and nonamplified patients. All hypothesis tests were conducted at the two-sided *P *= 0.05 level. For detailed description of statistical analyses, we refer to a previous report [42].

## Results

### Distribution of *CCND1 *amplification status and cyclin D_1 _protein staining categories

**Figure 1:**
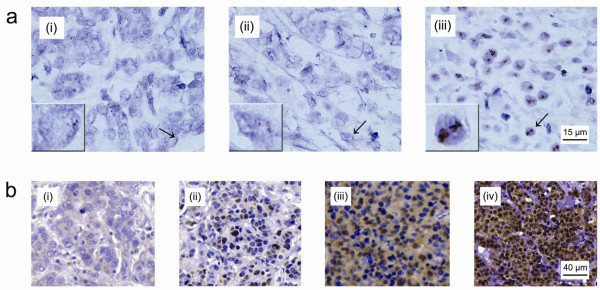
**Chromogenic *in situ *hybridisation and immunohistochemical staining of breast cancer samples**. **(a) **Two copies of the *CCND1 *gene represents nonamplified patients (i). A copy number of 3 to 8 copies was considered a gain (ii). Amplified tumours often exhibit a very high number of *CCND1 *gene copies (iii). **(b) **Low cytoplasmic staining without nuclear cyclin D_1 _expression (i). Low cyclin D_1 _expression in cytoplasm and low fraction-positive nuclei displaying weak staining intensity of most nuclei (ii). Intermediate cytoplasmic expression and fraction-positive nuclei, with nuclei showing moderate staining intensity (iii). High cytoplasmic and nuclear expression of cyclin D_1_, with nuclear fraction > 67% (iv).

In the ATAC trial, 5,880 hormone-receptor-positive breast cancer patients were randomly assigned to receive the monotherapy anastrozole or tamoxifen. For the TransATAC protocol, 1,868 patients from the monotherapy arms were initially included. In the present study, 627 patients were not assessable for *CCND1 *amplification status due to missing or damaged tissue cores, and 86 patients were excluded as they did not meet the study criteria of being ER-positive, leaving 1,155 patients assessable for *CCND1 *amplification status. Out of the patients with known amplification status, 1,054 (91.3%) exhibited nonamplified tumours and 101 (8.7%) were amplified (Figure 1a). High cyclin D_1 _cytoplasmic intensity was observed in 380 tumours (32.9%), high nuclear cyclin D_1 _intensity in 278 tumours (24.1%), and 190 tumours (16.5%) had > 67% nuclear fraction positivity as detailed in Additional file 1. *CCND1 *gene amplification was associated with a higher expression of nuclear cyclin D_1 _(*P *< 0.001) as well as a higher fraction of positive nuclei (*P *< 0.001), but was not significantly correlated to the amount of cytoplasmic protein (*P *= 0.063) (Table 1 and Figure 1b). Furthermore, positive correlations were observed between nuclear and cytoplasmic components of cyclin D_1 _protein expression.

**Table 1:** Associations between *CCND1 *amplification status, cyclin D_1 _protein expression and clinicopathological variables in all patients

All patients	Cyclin D_1 _cytoplasmic intensity (negative/low, intermediate, high)	Cyclin D_1 _nuclear intensity (negative/low, intermediate, high)	Cyclin D_1 _nuclear fraction (< 1%,1 to 9%, 10 to 32%, 33 to 67%, > 67%)	Tumour grade (well, moderate, poor)	Tumour size^a ^(mm)	Nodal status (negative, 1 to 3, > 3)	Ki67^a^
*CCND1 *amplification							
Nonamplified	12%, 52%, 36%	40%, 36%, 24%	10%, 24%, 27%, 24%, 15%	23%, 61%, 16%	18.0	70%, 23%, 7%	4.0
Amplified	6%, 48%, 46%	9%, 35%, 56%	0%, 6%, 18%, 32%, 44%	9%, 62%, 29%	19.0	64%, 26%, 10%	7.5
	Armitage (*P *= 0.063)	Armitage (*P *< 0.001)	Armitage (*P *< 0.001)	Armitage (*P *< 0.001)	Wilcoxon (*P *= 0.083)	Armitage (*P *= 0.341)	Wilcoxon (*P *< 0.001)
Cyclin D_1 _cytoplasmic intensity							
Negative/low		74%, 16%, 10%	23%, 33%, 26%, 11%, 4%	26%, 59%, 15%	17.5	68%, 24%, 8%	3.2
Intermediate		40%, 36%, 24%	10%, 24%, 27%, 25%, 14%	17%, 63%, 20%	19.0	64%, 34%, 2%	4.6
High		20%, 43%, 37%	3%, 16%, 25%, 28%, 28%	23%, 61%, 16%	17.0	74%, 20%, 6%	5.6
		Goodman = 0.44 (*P *< 0.001)	Goodman = 0.39 (*P *< 0.001)	Goodman = -0.06 (*P *= 0.238)	Cuzick trend (*P *= 0.010)	Goodman = -0.12 (*P *= 0.031)	Cuzick trend (*P *= 0.021)
Cyclin D_1 _nuclear intensity							
Negative/low			23%, 33%, 26%, 11%, 4%	24%, 62%, 14%	18.0	67%, 25%, 8%	2.9
Intermediate			1%, 12%, 41%, 36%, 10%	18%, 65%, 17%	18.0	68%, 25%, 7%	4.8
High			0%, 1%, 7%, 37%, 55%	17%, 59%, 24%	17.5	71%, 22%, 7%	6.3
			Goodman = 0.88 (*P *< 0.001)	Goodman = 0.16 (*P *< 0.001)	Cuzick trend (*P *= 0.553)	Goodman = -0.05 (*P *= 0.327)	Cuzick trend (*P *< 0.001)
Cyclin D_1 _nuclear fraction							
< 1%				18%, 68%, 14%	20.0	2%, 29%, 9%	2.8
1 to 9%				23%, 62%, 15%	18.0	68%, 25%, 7%	2.7
10 to 32%				24%, 59%, 17%	18.0	71%, 25%, 4%	4.4
33 to 67%				20%, 59%, 21%	18.0	70%, 21%, 9%	4.9
> 67%				12%, 68%, 20%	16.0	68%, 24%, 8%	5.9
				Cuzick trend (*P *= 0.005)	Spearman ρ = -0.04 (*P *= 0.162)	Cuzick trend (*P *= 0.694)	Spearman ρ = 0.22 (*P *< 0.001

Tests used: Armitage's trend test, Wilcoxon test, Goodman's test, Spearman correlation test and Cuzick test. ^a^Median value for each category.

### *CCND1 *gene amplification and patient prognosis

**Figure 2:**
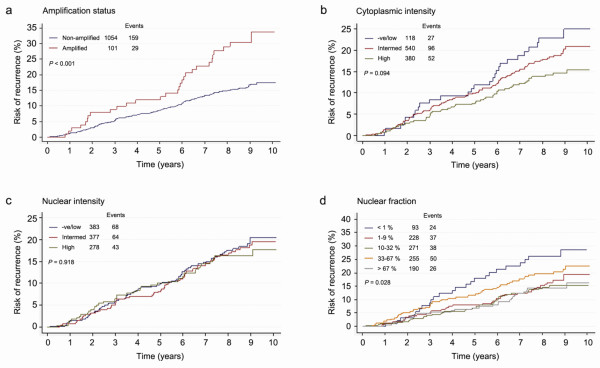
**Kaplan-Meier plots of recurrence risk over time in all patients**. **(a) **Risk of recurrence was increased for patients exhibiting *CCND1*-amplified breast cancers compared with nonamplified. **(b), (c) **No significant difference was observed between varying intensities of cytoplasmic or nuclear cyclin D_1_. **(d) **Patients showing a nuclear fraction of cyclin D_1 _lower than 1% had an increased risk of recurrence compared with higher expression.

Initially we studied the association between *CCND1 *amplification status and TTR. Survival plots showed that patients exhibiting *CCND1*-amplified tumours had an increased risk of recurrence compared with patients showing nonamplified tumours (HR = 2.04; 95% CI, 1.37 to 3.03; χ_1_^2 ^= 10.51; *P *< 0.001, univariate) (Figure 2a). Even when adjusting for the effects of tumour size, nodal status, grade (central) and Ki67 expression, amplification of *CCND1 *was significantly associated with an increased risk of recurrence (HR = 1.61; 95% CI, 1.08 to 2.41; *P *= 0.03) (Table 2).

**Table 2:** Cox proportional Hazards models for estimating the effect on time to recurrence

	Univariate			Multivariate		
	
	HR (95% CI)	χ_1_^2^	*P *value	HR (95% CI)	χ_1_^2^	*P *value
All patients						
CCND1, amplified vs. nonamplified	2.04 (1.37 to 3.03)	10.51	< 0.001	1.61 (1.08 to 2.41)	4.86	0.030
Cyclin D_1 _cytoplasmic intensity, high vs. remainder	0.71 (0.51 to 0.98)	4.43	0.039	0.78 (0.56 to 1.09)	2.15	0.143
Cyclin D_1 _nuclear intensity, high vs. remainder	0.91 (0.64 to 1.28)	0.30	0.588	0.85 (0.59 to 1.21)	0.84	0.360
Cyclin D_1 _nuclear fraction, 1 to 100% vs. < 1%	0.58 (0.38 to 0.90)	5.28	0.014	0.60 (0.39 to 0.92)	4.75	0.030
Nonamplified patients						
Cyclin D_1 _cytoplasmic intensity, high vs. remainder	0.57 (0.39 to 0.82)	9.57	0.003	0.64 (0.44 to 0.95)	5.21	0.022
Cyclin D_1 _nuclear intensity, high vs. remainder	0.88 (0.60 to 1.32)	0.38	0.545	0.84 (0.55 to 1.26)	0.76	0.382
Cyclin D_1 _nuclear fraction, 1 to 100% vs. < 1%	0.52 (0.34 to 0.81)	7.29	0.004	0.54 (0.35 to 0.85)	6.36	0.012

The χ_1_^2 ^value is based on the likelihood ratio test with the associated *P *values. Multivariate analyses were adjusted for tumour grade, tumour size, nodal status and Ki67. CI, confidence interval; HR, hazard ratio.

### Cyclin D_1 _protein expression and patient prognosis

**Figure 3:**
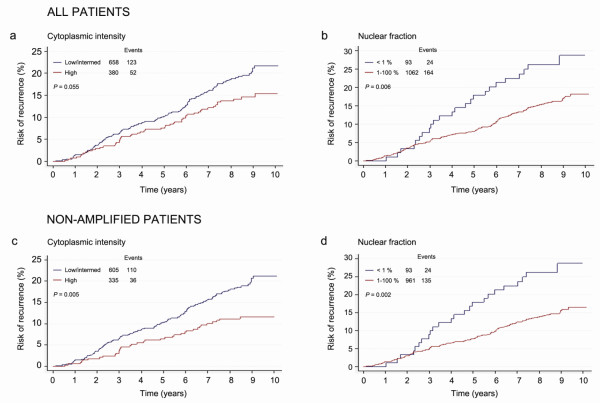
**Predicted risk of recurrence over time based on cytoplasmic intensity and nuclear fraction of cyclin D_1_, comparing two subgroups**. **(a) **In all patients, no significant difference in recurrence risk was observed between low and high cytoplasmic expression. **(b) **Patients showing a nuclear fraction of less than 1% positive cyclin D_1 _nuclei had an increased risk of recurrence compared with a fraction of 1 to 100%, in all patients. **(c) **In patients exhibiting nonamplified tumours, high cytoplasmic expression was associated with a reduced risk of recurrence. **(d) **A fraction of cyclin D_1_-positive nuclei lower than 1% was associated with a higher recurrence risk also in patients showing nonamplified tumours.

We next investigated how cyclin D_1 _protein localisation and expression was related to TTR. There was no significant difference in TTR with relation to cytoplasmic cyclin D_1 _(Figure 2b) or cyclin D_1 _nuclear intensity (Figure 2c and Table 2). Surprisingly, a greater fraction of cyclin D_1_-positive nuclei was associated with longer TTR (HR = 0.60; 95% CI, 0.39 to 0.92; *P *= 0.03) when adjusted for the effects of tumour size, nodal status, grade and Ki67 expression (Figures 2d, 3b and Table 2). Also, when subcategorising patients into high versus lower subgroups of cytoplasmic cyclin D_1 _there was a trend towards a significant difference in TTR (*P *= 0.055; univariate, HR = 0.71; 95% CI, 0.51 to 0.98; *P *= 0.039) (Figure 3a and Table 2). When focusing on the non-amplified breast cancer samples, high cytoplasmic cyclin D_1 _protein expression was indeed associated with a better outcome (*P *= 0.005) (Figure 3c and Table 2). The data further indicated that the lowest fraction of cyclin D_1_-positive nuclei (< 1%) was associated with shorter TTR compared with subgroups of higher percentage positive nuclei, as illustrated in Figure 3b, d. Unfortunately, the number of *CCND1-*amplified cases was too low to analyse survival according to nuclear protein expression; that is, this subgroup contained no cases exhibiting a nuclear protein expression < 1%.

### Interactions of cyclin D_1 _and treatment, nodal status and Ki67

**Figure 4:**
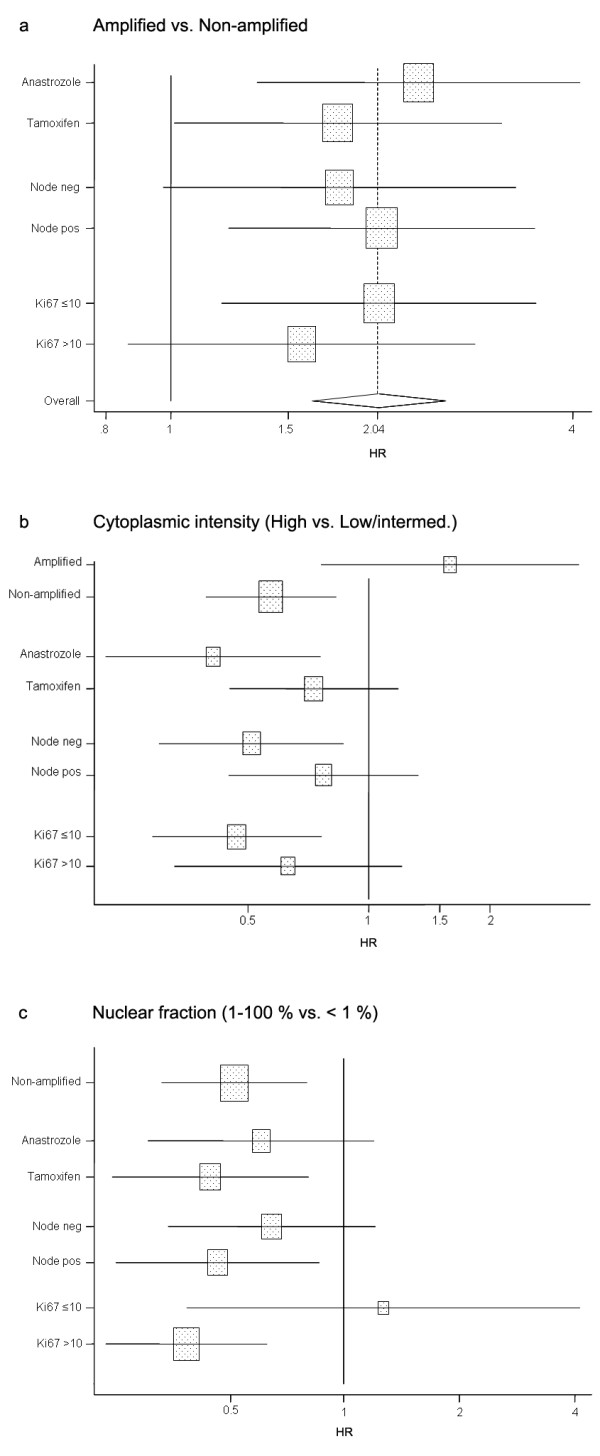
**Forest plot showing the effect of *CCND1 *amplification, cytoplasmic and nuclear cyclin D_1 _expression**. **(a) **Amplified against nonamplified breast cancers in the subgroups of treatment, nodal status and Ki67. **(b) **High cytoplasmic intensity against low or intermediate intensity. **(c) **Nuclear fraction of 1 to 100% against < 1% positive nuclei. Reference population for all subgroups was nonamplified patients (except for amplification status). HR, hazard ratio; neg, negative; pos, positive.

Based on previous reports indicating that amplification of the *CCND1 *gene and cyclin D_1 _overexpression might be associated with tamoxifen resistance or detrimental effects, we wanted to elucidate whether this could be further clarified in the patient cohort of the present study. The subgroup of patients treated with tamoxifen included 571 cases, and the anastrozole-treated subgroup included 584 patients. For *CCND1 *amplification status there was no significant difference in TTR between anastrozole-treated and tamoxifen-treated patients (Figure 4a). Moreover, for nonamplified cases there was no difference according to nodal status or Ki67 levels between the treatment arms. For cytoplasmic cyclin D_1 _expression there was an association in TTR according to amplification status (HR = 2.8; 95% CI, 1.2 to 6.4 for the interaction) (Figure 4b). In nonamplified breast cancers, however, no significant difference in TTR was observed for treatment (HR = 1.8; 95% CI, 0.8 to 3.9 for the interaction), nodal status (HR = 1.5; 95% CI, 0.7 to 3.2) or Ki67 levels (HR = 1.3; 95% CI, 0.6 to 3.0) (Figure 4b). Finally, for the nuclear fraction we observed an association in Ki67 levels for TTR (HR = 3.5; 95% CI, 1.0 to 12.3 for the interaction) (Figure 4c).

### Cyclin D_1_, *CCND1 *and clinicopathological data

The *CCND1 *amplification status was positively correlated to tumour grade (*P *< 0.001) and proliferation (defined as Ki67 expression) (*P *< 0.001), but not to nodal status or tumour size (Table 1). Cytoplasmic cyclin D_1 _expression was inversely correlated to tumour size (all patients; *P *= 0.01) and nodal status (*P *= 0.031), and was positively correlated to proliferation (*P *= 0.021). Both nuclear staining intensity of cyclin D_1 _and fraction-positive nuclei were associated with higher grade (*P *< 0.001 and *P *= 0.005 respectively) and higher proliferation rate (both *P *< 0.001).

### Cyclin D_1_, proliferation and time to recurrence

**Figure 5:**
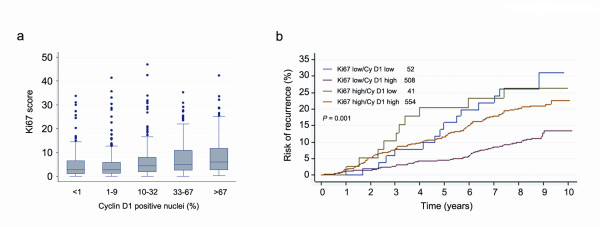
**Recurrence risk over time based on the expression of Ki67 and cyclin D_1_**. **(a) **Expression of Ki67 increased with increasing percentage of cyclin D_1_-positive nuclei in the breast tumours. **(b) **Kaplan-Meier plot showing that the combination of high Ki67 and low cyclin D_1 _expression was associated with high risk of recurrence (green). Patients exhibiting a high fraction of cyclin D_1_-positive tumour cell nuclei in concurrence with low Ki67 expression showed the lowest risk of recurrence (red).

Ki67 expression is a common marker used to analyse the proliferation rate in tumour samples, and high proliferation is linked to a more aggressive tumour phenotype. Surprisingly, despite a positive correlation between nuclear cyclin D_1 _expression and Ki67 expression (Table 1 and Figure 5a), high expression of nuclear cyclin D_1 _was associated with an improved TTR compared with low expression. To further illustrate how a combined proliferation and cyclin D_1 _assessment would be linked to TTR we subdivided patients according to Ki67 expression and nuclear cyclin D_1 _status. Patients with tumours exhibiting low Ki67 expression in association with 1 to 100% cyclin D_1_-positive nuclei (high) were associated with a considerably lower risk of recurrence (*P *< 0.001) (Figure 5b) compared with the other subgroups. The subgroups of low cyclin D_1 _include quite low patient numbers and hence the results should be interpreted with caution, but these results suggest that the expression of cyclin D_1 _affects disease outcome independently of proliferation status.

## Discussion

Amplification of the *CCND1 *gene has been associated with a poor patient outcome in previous studies [19,26], whilst controversy regarding overexpression of cyclin D_1 _protein in relation to patient survival still exists. Cyclin D_1 _has been reported to be a prognostic marker in invasive breast cancer and has been associated with both a less aggressive ER-positive phenotype [20,22] and also with an adverse clinical outcome [18]. These conflicting findings can potentially be explained by the low patient numbers analysed and/or methodological discrepancies. To clarify the importance of cyclin D_1 _in breast cancer we therefore analysed the expression of cyclin D_1 _in different subcellular localisations, using a previously validated antibody [36], as well as the gene amplification status by the well-established CISH technique in a large, well-characterised randomised patient cohort including more than 1,000 patients with ER-positive breast cancers. Our data support studies indicating that low cyclin D_1 _protein expression as well as *CCND1 *amplification are linked to tumour aggressiveness and increased risk of disease recurrence in ER-positive postmenopausal breast cancer [23,26]. Similar findings have been observed for HER2, where both high expression linked to amplification and low expression are linked to poor outcome [43].

Amplification of *CCND1 *was observed in 8.7% of the tumours, which is slightly lower than the frequency of 10 to 15% generally reported, even though some groups have demonstrated a lower percentage of *CCND1*-amplified tumours [15,44,45]. The slightly lower fraction of *CCND1*-amplified cases may be due to all patients being ER-positive or due to methodological differences and different cutoff points for defining amplification between studies. In addition, the use of TMAs has certain limitations; however, this technique is indispensable when analysing large patient materials and is today a well-accepted approach for large-scale tumour sample analysis. In agreement with previous studies, gene amplification of *CCND1 *was associated with an overall adverse clinical outcome. The observed positive correlation between nuclear cyclin D_1 _expression and tumour grade and proliferation suggests a link between cyclin D_1 _and aggressive disease. In contrast, both higher nuclear expression and high cytoplasmic expression of cyclin D_1 _was instead associated with a decreased recurrence risk. Despite a positive association between cyclin D_1 _protein and *CCND1 *amplification status, both low nuclear fraction of cyclin D_1 _and *CCND1 *amplification were linked to earlier disease recurrence independently of other clinicopathological parameters - hence both factors serve as prognostic markers in endocrine-treated, ER-positive postmenopausal breast cancer. In patients not displaying *CCND1 *amplification, cytoplasmic expression of cyclin D_1 _was also an independent marker for longer TTR, indicating that the true prognostic value of cyclin D_1 _protein expression may be obscured by the *CCND1*-amplified cases: the clinicopathological significance of cyclin D_1 _expression might thus be best considered separately for amplified and nonamplified cases.

Apart from the role as a prognostic marker, cyclin D_1 _has been proposed as a predictive factor for tamoxifen response, as illustrated by poor clinical outcome in patients with ER-positive tumours with high cyclin D_1 _expression treated with tamoxifen [46]. These findings together with numerous experimental reports [33-35] support that cyclin D_1 _overexpression might abrogate the response to tamoxifen, as previously reported by our group and others [21,46]. Our earlier discoveries have nevertheless been made in cohorts where patients were randomly assigned to receive either no adjuvant treatment or to receive tamoxifen [21,36]. In the present study we compared the two endocrine therapies anastrozole and tamoxifen in relation to disease recurrence and cyclin D_1 _status, but there were no untreated control patients. There was no significant difference in treatment response between these two adjuvant therapies by stratification for cyclin D_1 _status, indicating that cyclin D_1 _is not a predictive marker for differences in response to anastrozole versus tamoxifen. No conclusions can be drawn, however, regarding cyclin D_1 _as a marker for general endocrine treatment resistance, since no untreated patients were available for analysis within the TransATAC study. Moreover, differences in tamoxifen response in relation to cyclin D_1 _in postmenopausal versus premenopausal breast cancer might exist. Our previous study reporting a potential unfavourable effect of tamoxifen included premenopausal patients exclusively, whereas this study focused exclusively on postmenopausal breast cancer cases and has shown no detrimental effect since the results for the two endocrine therapies were similar.

The relationship between cyclin D_1_, proliferation and prognosis is quite complex, with a positive correlation between cyclin D_1 _and Ki67 - Ki67 is associated with shorter TTR, while cyclin D_1 _is associated with longer TTR. Patients showing low expression of Ki67 had a longer TTR with the highest levels of cyclin D_1 _expression, whereas patients exhibiting higher levels of Ki67 had the shortest TTR with the lowest levels of cyclin D_1 _expression. Multivariate analysis identified the fraction cyclin D_1_-positive nuclei as a predictor of outcome independently of other clinicopathological parameters such as Ki67. These results suggest that, irrespective of proliferation status, intermediate to high expression of cyclin D_1 _results in a prolonged TTR in ER-positive, postmenopausal breast cancer patients. Similar results were observed in our previous study of randomised material from premenopausal breast cancer patients [47]. The relationship between cyclin D_1_, proliferation and prognosis hence seems to be complex, and this could in part be explained by potential additional functions for cyclin D_1 _unrelated to proliferation control, as well as co-amplification and co-deletion of specific genes on chromosome 11q13, the locus harbouring *CCND1*.

## Conclusions

This study confirms that the cyclin D_1 _status provides independent prognostic information regarding ER-positive, postmenopausal breast cancers, supporting its emerging role as a biomarker that might be useful in the clinic. Our results demonstrate that high expression of cyclin D_1 _was associated with a reduced risk of recurrences, whereas amplification of the *CCND1 *gene was linked to an aggressive disease. Finally, no difference in response to anastrozole compared with tamoxifen was observed according to expression of cyclin D_1 _gene amplification or protein expression.

## Abbreviations

ATAC: Arimidex, Tamoxifen, Alone or in Combination; CI: confidence interval; CISH: chromogenic *in situ *hybridisation; ER: oestrogen receptor; HR: hazard ratio; TMA: tissue microarray; TTR: time to recurrence.

## Competing interests

JC has acted as a consultant and/or on advisory boards for AstraZeneca and received commercial research grants from AstraZeneca. AH has acted as a consultant and/or on advisory boards for AstraZeneca, GSK, Pfizer, Roche and Amgen, and has received honoraria from AstraZeneca. MD receives paid advisory boards and research funding from each of Novartis, AstraZeneca and Roche. The remaining authors declare that they have no competing interests.

## Authors' contributions

KL carried out the immunohistochemical and CISH assessments, participated in the study design and drafted the manuscript. MB assisted with evaluation of the immunohistochemistry. SP performed the statistical analyses. JC designed the clinical trial, assembled trial data, and planned and performed the statistical analyses. JS assembled trial data. LZ assembled trial data. AH designed the clinical trial and collected clinical data. MD designed the clinical trial and collected clinical data, and initiated and conceived the current study. GL initiated and conceived the study, supervised interpretation of the data, took part in the immunohistochemical assessments and evaluation of CISH, and helped to draft the manuscript. All authors took part in data interpretation, writing the report and approval of the final version of the manuscript.

## Supplementary Material

Additional file 1 **Supplementary **Table 1**showing the distribution of cyclin D_1 _staining categories**. Click here for file
