# Supplementary material for: Effects of cyclin D1 gene amplification and protein expression on time to recurrence in postmenopausal breast cancer patients treated with anastrozole or tamoxifen: a TransATAC study
Source: Breast Cancer Res. 2012 Apr 4;14(2):R57. doi: 10.1186/bcr3161 (PMC3446392; doi:10.1186/bcr3161)
Supplement: Additional file 1 — Supplementary Table 1showing the distribution of cyclin D1 staining categories. [file bcr3161-S1.DOC]

Supplementary table 1. Distribution of cyclin D1 staining categories.

|  | ***All patients*** | ***Non-amplified patients*** |
| --- | --- | --- |
| ***CCND1*** **Amplification**  Non-amp  Amp | 1054 (91.3 %)  101 (8.7 %) | 1054 (100 %)  - |
| **Cyclin D1 Cytopl int**  -ve/low  Interm  High | 118 (10.2 %)  540 (46.8 %)  380 (32.9 %) | 112 (10.6 %)  493 (46.8 %)  335 (31.8 %) |
| **Cyclin D1 Nuclear int**  -ve/low  Interm  High | 383 (33.2 %)  377 (32.6 %)  278 (24.1 %) | 374 (35.5 %)  343 (32.5 %)  223 (21.2 %) |
| **Cyclin D1 Nuclear fract**  < 1 %  1 % - 9 %  10 % - 32 %  33 % - 67 %  > 67 % | 93 (8.1 %)  228 (19.7 %)  271 (23.5 %)  255 (22.1 %)  190 (16.5 %) | 93 (8.8 %)  222 (21.1 %)  253 (24.0 %)  224 (21.3 %)  147 (13.9 %) |
